# Teaching Methods Utilized During Medical Resuscitations in an Academic Emergency Department

**DOI:** 10.5811/westjem.2018.5.37521

**Published:** 2018-06-11

**Authors:** Lori A. Weichenthal, Rawnie Ruegner, Stacy Sawtelle, Danielle Campagne, Crystal Ives, James Comes

**Affiliations:** University of California, San Francisco-Fresno, Department of Emergency Medicine, Fresno, California

## Abstract

**Introduction:**

One important skill that an emergency medicine trainee must learn is the resuscitation of the critically ill patient. There is research describing clinical teaching strategies used in the emergency department (ED), but less is known about specific methods employed during actual medical resuscitations. Our objective was to identify and describe the teaching methods used during medical resuscitations.

**Methods:**

This was a prospective study involving review of 22 videotaped, medical resuscitations. Two teams of investigators first each reviewed and scored the amount and types of teaching observed for the same two videos. Each team then watched and scored 10 different videos. We calculated a Cohen’s kappa statistic for the first two videos. For the remaining 20 videos, we determined means and standard deviations, and we calculated independent two-tailed t-tests to compare means between different demographic and clinical situations.

**Results:**

The Cohen’s kappa statistic was K=0.89 with regard to number of teaching events and K=0.82 for types of teaching observed. Of the resuscitations reviewed, 12 were in coding patients. We identified 148 episodes of teaching, for an average of 7.4 per resuscitation. The amount of teaching did not vary with regard to whether the patient was coding or not (p=0.97), nor based on whether the primary learner was a junior or senior resident (p=0.59). Questioning, affirmatives and advice-giving were the most frequently observed teaching methods.

**Conclusion:**

Teachers use concise teaching methods to instruct residents who lead medical resuscitations. Further research should focus on the effectiveness of these identified strategies.

## INTRODUCTION

The emergency department (ED) is a rich learning environment for learners, abounding with undifferentiated, complicated and critically ill patients. Clinical teaching in the ED not only provides learners with the opportunity to improve their fund of knowledge, but perhaps more importantly it impacts their clinical acumen, procedural skills and development as professional healers.^1^–^3^ Despite the wealth of learning opportunities in the ED, traditional teaching methods may not be as effective in this clinical environment, especially during an acute resuscitation where care of the seriously ill or injured patients must be prioritized over all other tasks.^4^,^5^ To be effective in these high-stakes situations, successful teachers need to have an efficient and dynamic set of teaching tools.^5^

Multiple studies have been published on effective bedside teaching in the ED and other environments where time is limited.^4^–^8^ Other research describes teaching residents during critical resuscitations; however, the majority of these are done in the setting of simulation. 9–11 Less is known about what teaching methods are used during actual medical resuscitations in the ED.

Prior to determining the most effective teaching strategies to use during medical resuscitations, it is necessary to establish what types of teaching methods are currently being used. One study by Grall et al. attempted to codify the types of teaching observed in an academic ED. They discovered that in addition to previously described teaching methods, such as questioning and limited teaching points, up to six previously undescribed strategies were used by teachers in this setting, including advice-giving and affirmatives (Table 1).^8^ This study, however, was not limited to the environment of an acute medical resuscitation, and the type of teaching strategies used in this more dynamic situation may differ from those used in other situations in the ED. The purpose of this study was to further elucidate the type of strategies that are used to teach learners during medical resuscitations in an academic ED.

**Population Health Research Capsule**

What do we already know about this issue?

To teach in the highly dynamic environment of the emergency department (ED) requires efficient teaching tools that challenge the learner but also help to direct care.

What was the research question?

What teaching strategies are used to teach learners during medical resuscitations in the ED?

What was the major finding of the study?

Concise methods are used to teach during medical resuscitations, including questioning, affirmatives and advice-giving.

How does this improve population health?

This study provides the groundwork to further investigate the efficacy of teaching during resuscitations. Improving the teaching provided during these events could improve the care of the critically ill.

## METHODS

We conducted a prospective, observational, primarily descriptive study involving review of video-recorded medical resuscitations from May to September 2016 in the ED of Community Regional Medical Center (CRMC) in Fresno, California. This study was approved by the CRMC institutional review board, and a waiver for informed consent was obtained. A policy on the use of videotaped resuscitations exists at CRMC explaining its use for quality improvement, education and research. Learners, faculty and nurses have the opportunity to opt out of being videotaped.

CRMC is the only Level I trauma center and burn center for the Central Valley of California. The ED cares for 115,000 patients per year and is home to a four-year emergency medicine (EM) residency training program with 40 residents. A resident in the second post-graduate year (PGY-2) or higher and a faculty member are primarily assigned to cover the high acuity medical area of the ED. Residents are responsible for the assessment and care of the patients and present all patients to faculty. During the majority of medical resuscitations, both the resident and faculty are present during the initial assessment and treatment. Besides the resident and faculty running the resuscitation, other teachers and learners may be in the room including medical students, residents, and faculty who present from other areas of the ED during a resuscitation to assist or learn from the situation.

Critically ill patients arriving via ambulance are announced overhead as a medical resuscitation or as a code blue. An attempt is made to place all of these patients initially into a designated resuscitation room with video- recording capabilities. When the patient arrives, the system is activated and the resuscitation is recorded.

Beginning in May 2016, 22 consecutive, video-recorded medical resuscitations were collected. The six investigators, who on average have 11 years of experience as clinical educators, worked in two teams of three. All investigators have roles in the EM program that involve evaluation of residents and faculty.

We designed a structured observation form, using the 14 teaching methods described in the Graff et al. study. The form also had areas to collect demographics and situational information (Table 2). Prior to the initiation of the study, all investigators met to be trained on the use of this structured form and to be provided examples of each type of teaching strategy. Opportunity existed to discuss and clarify the different classifications and definitions of the teaching methods. Then each team of three, in separate locations, watched the same two test videos and scored their observations on the form. These data from the pilot observations were then used to determine interrater reliability.

Each team of three was then assigned 10 videotapes to observe for a total of 20 separate resuscitations. While watching the videos, the investigators considered not only the interaction between the faculty and the primary resident leading the resuscitation, but also among other learners and teachers in the room. Each video, thus, had several possible learner interactions, and each type of interaction was recorded separately. Team members completed the structured observation form individually and reviewed their results at the end of each video. Discrepant results were discussed, and relevant video segments were reviewed until consensus was reached.

Data from the two pilot observations were entered into an Excel 2013 spreadsheet (Microsoft, Redmond, WA), and we calculated a Cohen’s kappa statistic to determine interrater reliability between the two groups of investigators with regard to the number and types of teaching documented. Data from the 20 observation forms were then entered into an Excel 2013 spreadsheet, where means and standard deviations were calculated when appropriate. We calculated independent, two-tailed t-tests to compare the means between different demographic (e.g., type of teacher and learner) and clinical situations (e.g., “coding patients,” pulseless patients receiving active cardiopulmonary resuscitation [CPR] vs. “non-coding patients,” unstable patients with a pulse not undergoing CPR).

## RESULTS

The Cohen’s kappa statistic comparing the two groups of investigators for the test video recordings was K=0.89 with regard to number of teaching events and K=0.82 for types of teaching documented, suggesting a high degree of interrater reliability between the two groups. We identified 148 teaching episodes during the 20 resuscitations for an average of 7.4 per case (range 1–10). Sixty-five of these teaching episodes were between faculty and senior residents (PGY-3 or -4), sixty-two between faculty and junior residents (PGY-2), four faculty to medical student, eight senior resident to junior resident, and nine resident to medical student.

Of the resuscitations reviewed, 12 were coding patients and the rest were non-coding patients requiring urgent resuscitation. All coding patients had an assigned Emergency Severity Score (ESI) of one. Five of the non-coding patients had an ESI of one and the remaining three had an ESI of 2. Five of the non-coding patients presented with altered level of consciousness, three of which required intubation. Two other patients presented after return of spontaneous circulation in the field and also required intubation. The remaining patient presented with hypotension and bradycardia.

The quantity of teaching did not vary significantly with regard to the clinical status of the patient, coding vs. non-coding (7.42 vs. 7.38, p=0.97); however, in non-coding patients the frequency of teaching was greater in the more critically ill patients (ESI=1) (9.6 vs. 4.3, p=0.002). The amount of teaching by faculty of the primary resident running the resuscitation did not significantly change based on whether the learner was a junior (PGY-2) or senior resident (PGY-3 or 4) (5.64 vs. 5.0, p=0.59).

The most common methods of teaching used between the faculty and primary resident during the resuscitations, both codes and non-codes, were questioning, affirmatives and advice-giving (Table 3). Questioning was the most-used technique both in coding and non-coding patients; however, advice-giving and bedside teaching were the next most-used methods in the setting of a coding patient, whereas affirmatives and limited teaching points were more common in situations involving non-coding patients (Table 4). The teaching methods most commonly used (questioning, affirmatives and limited teaching points) were the same when comparing the more critically ill non-coding patients (ESI=1) to the less critically ill (ESI=2).

Questioning was the most frequently used teaching method by faculty for both junior and senior residents and advice-giving was used equally among these learners. Faculty, however, were more likely to use limited teaching points with junior residents, whereas with senior residents they frequently relied on affirmatives (Table 5).

On average, two learners were present during each resuscitation (range 1–4). Interns and medical students in the room were present mainly as observers or performing procedures. The most common teaching methods used for these learners were limited teaching points, teaching at bedside, and mini-lecture (Table 5). Reflective modeling or pattern recognition were not observed during these resuscitations.

## DISCUSSION

Grall and colleagues published an observational study of teaching in the ED involving patients of a range of different levels of acuity. They found that questioning, advice-giving and limited teaching points were the most frequently used teaching methods.^8^ This study sought to establish which methods of teaching are most commonly used during resuscitation, in an effort to guide further evaluations of effectiveness.

Questioning is heavily used during medical resuscitations in both coding and non-coding patients. Questioning in coding patients tended to be more focused on directing care of the patient (e.g., “What medication should you give next?”), whereas in the non-coding patient there were more examples of questioning used to more deeply probe the knowledge of the learner (e.g., “What are the potential causes of bradycardia in this patient?”)

In coding patients, the next two most-common teaching methods were advice-giving and bedside teaching. Advice-giving, first described by Grall and his colleagues, is a rapid way for the teacher to guide the resident in the next stages of care (e.g., “I would intubate the patient next’) in the time-sensitive situation of a coding patient.^8^ Bedside teaching in this situation was most commonly procedurally based (e.g., bedside ultrasound, placement of central line).

In non-coding patients, after questioning, affirmatives and limited teaching points were the next most frequent teaching methods used. Limited teaching points may be more likely to be used in the setting of the non-coding patient because there is more time to do directed teaching when compared with the more time-sensitive scenario of the coding patient.

Questioning, the most frequently used teaching method for both senior and junior residents, was identified by Heidenreich and Ramani to be both effective and efficient.^3^,^12^, ^13^ The intent of the questions varied from narrow and specific, seeking to yield information for further consideration or to assess the learner’s knowledge base (e.g., “The patient has been down for how long?”, “What dose of amiodarone would you give?”) to broader questions used to stimulate the learner’s critical thinking and problem-solving skills or to guide patient management (e.g.; “What other medications would you consider giving at this point?”; “What are the treatable causes of pulseless electrical activity?”).

Advice-giving, first described as a teaching method by Grall and colleagues, was also used by attendings in this study to teach both junior and senior residents who had primary responsibility for the care of the patient being resuscitated. The teacher provides advice to the learner (e.g., “I would use the bedside ultrasound to assess for cardiac activity at this point”; “I would administer broad spectrum antibiotics”). Advice-giving may be an efficient method of teaching because it is immediately applicable and builds on prior knowledge of the learner.^8^

For senior residents, affirmatives were one of the top three teaching methods used. Affirmatives were also first described by Grall et al. Affirmatives can be verbal or non-verbal and serve to inform learners that the teacher agrees with their plan or cognitive process.^8^ Affirmatives may be more frequently used with senior learners because the teacher is more confident of their knowledge base and skills and only feels the need to assure the resident that he or she is on task.

For junior residents, limited teaching points was one of the top three teaching methods used. Limited teaching points was previously described by Heidenreich and Bandiera as a valid teaching method.^12^,^14^ Limited teaching points usually relates to a specific aspect of the patient’s care (e.g., discussing the pros and cons of using etomidate vs. ketamine in a septic patient) and are concise and specific. They may be more commonly used for junior residents because the teacher feels there are knowledge gaps that need to be addressed in a rapid manner during the resuscitation.

Limited teaching points were also used for interns and medical students, but more in-depth teaching methods including teaching at bedside (usually procedurally based) and mini-lectures on a topic related to the patient’s care (“Let’s go over the 5 H’s and T’s of PEA”) also were observed. The teaching of these learners was usually conducted by a faculty member or resident who was not primarily responsible for the resuscitation, but who had arrived to help. The peripheral role of interns and medical students during critical resuscitations at our institution might be the cause of the observed differences in how they are taught. This study helps to establish which types of teaching occur during medical resuscitations in both coding and non-coding patients. We identified several methods used by teachers in these time-sensitive cases that have been previously validated as both efficient and effective. This study, however, did not directly address the efficiency of these teaching methods.

## LIMITATIONS

There are several potential limitations to our study. The Kappa statistic to determine inter-rater agreement between the two groups that watched the videos was only calculated for the two pilot videos that were observed by both groups. After the two pilot videos, the two groups watched different videos, so no Kappa statistic could be calculated for the 20 videos included in the study; however, given that there was good inter-rater reliability with the two test videos, it is inferred that this reliability would continue throughout the other observations.

Only the first 20 resuscitations recorded during the study period were reviewed. Other videos may have revealed different teaching methods, although this cohort is likely a good representation and supports much of the earlier work by Grall and his colleagues. It is also possible that occasionally teaching that occurred prior to the start of the resuscitation were missed because the record button on the video camera was not pressed early enough. In addition, there was likely teaching surrounding the resuscitations that occurred between the resident and faculty that occurred later, off camera. It was not our goal to capture this interaction, however, as we sought to identify only teaching happening during the resuscitation effort itself.

Because all team members knew that the resuscitations were being recorded, this may have impacted the degree of teaching due to the Hawthorne effect. While we assessed the types of teaching during critical medical resuscitations and code situations, we did not assess the effectiveness of these teaching methods nor did we assess if these teaching methods improved resident learning outcomes; these are possible directions for future studies.

## CONCLUSION

Teachers use a variety of concise teaching methods to instruct residents who lead medical resuscitations. More in-depth teaching strategies are used for more-junior learners in the room. Further research should focus on the effectiveness of these identified strategies.

## Figures and Tables

**Table 1:** Teaching methods, previously reported and newly described, with definitions (adapted from Grall et al. emergency medicine teaching methods).

Method	Definition
Methods previously described in the literature	
Questioning	Challenges resident using questions; assesses resident’s knowledge with questions
Limited teaching points	Focused teaching on 1–2 key concepts
Bedside teaching	Traditional bedside teaching in the patient’s presence
Problem-oriented learning	Encourages learning from specific patient problems or management issues
Reflective modeling	Uses reflection on own practice to teach; explains own thought processes
Pattern recognition	Requests chief complaint and presumptive diagnosis before hearing case
Priming	Orients and focuses resident just prior to seeing patient
Feedback	Describes specific behaviors that were effective or need improvement
Newly described methods	
Advice giving	Gives advice on aspects of patient care
Patient updates	Resident gives update on patient information and attending provides reassurance
Affirmatives	Short affirmatives or nods to let learners know they are on the right track
Information sharing	Attending shares further information they have discovered independently
Role modeling	Demonstrates the role of an emergency physician with learner observing
Mini-lecture	Provides short lectures focused on one topic

**Table 2:** Coding categories and demographic data on observation form.

Coding categories Questioning Limited teaching points Teaching at bedside Problem-oriented learning Reflective modeling Pattern recognition Priming Feedback Advice giving Patient updates Affirmatives Information sharing Role modeling Mini-lecture Demographic data collected Patient status (coding vs. non-coding, presenting presentation for non-coding) Assigned emergency severity score Teacher’s level of training (faculty, fellow, senior resident) Learner’s level of training (PGY1–4, medical student) Number of learners in the resuscitation room

*PGY*, post-graduate year.

**Table 3:** Frequency of teaching methods during medical resuscitations n=148 (%).

Teaching method applied	n (%)
Questioning	51 (34)
Affirmatives	23 (16)
Advice giving	18 (12)
Limited teaching points	16 (11)
Teaching at bedside	13 (9)
Information sharing	10 (7)
Patient updates	6 (4)
Priming	3 (2)
Feedback	3 (2)
Role modeling	2 (1)
Mini-lecture	2 (1)
Problem-oriented learning	1 (1)
Reflective modeling	0(0)
Pattern recognition	0(0)

**Table 4:** Comparison of top three teaching methods used based on status of patient.

Status of patient	Most common methods (%)
Coding	Questioning (39) Advice giving (13) Bedside teaching (10)
Non-coding	Questioning (27) Affirmatives (27) Limited teaching points (15)

**Table 5:** Comparison of top three faculty teaching methods used based on level of learner.

Learner level	Most common methods (%)
Senior resident (PGY-3 or 4)	Questioning (35) Affirmatives (20) Advice giving (12)
Junior resident (PGY-2)	Questioning (40) Limited teaching points (15) Advice giving (11)
Interns and medical students	Limited teaching points (38) Teaching at bedside (24) Mini-lecture (10)

*PGY*, post-graduate year.
